# Microbial community dynamics in the forefield of glaciers

**DOI:** 10.1098/rspb.2014.0882

**Published:** 2014-11-22

**Authors:** James A. Bradley, Joy S. Singarayer, Alexandre M. Anesio

**Affiliations:** 1 School of Geographical Sciences, University of Bristol, Bristol BS8 1SS, UK; 2 Department of Meteorology, University of Reading, Reading RG6 6BB, UK

**Keywords:** deglaciated forefield soils, chronosequence, microbial succession, colonization, modelling, nutrient cycling

## Abstract

Retreating ice fronts (as a result of a warming climate) expose large expanses of deglaciated forefield, which become colonized by microbes and plants. There has been increasing interest in characterizing the biogeochemical development of these ecosystems using a chronosequence approach. Prior to the establishment of plants, microbes use autochthonously produced and allochthonously delivered nutrients for growth. The microbial community composition is largely made up of heterotrophic microbes (both bacteria and fungi), autotrophic microbes and nitrogen-fixing diazotrophs. Microbial activity is thought to be responsible for the initial build-up of labile nutrient pools, facilitating the growth of higher order plant life in developed soils. However, it is unclear to what extent these ecosystems rely on external sources of nutrients such as ancient carbon pools and periodic nitrogen deposition. Furthermore, the seasonal variation of chronosequence dynamics and the effect of winter are largely unexplored. Modelling this ecosystem will provide a quantitative evaluation of the key processes and could guide the focus of future research. Year-round datasets combined with novel metagenomic techniques will help answer some of the pressing questions in this relatively new but rapidly expanding field, which is of growing interest in the context of future large-scale ice retreat.

## 1. Introduction

**Figure 1.**
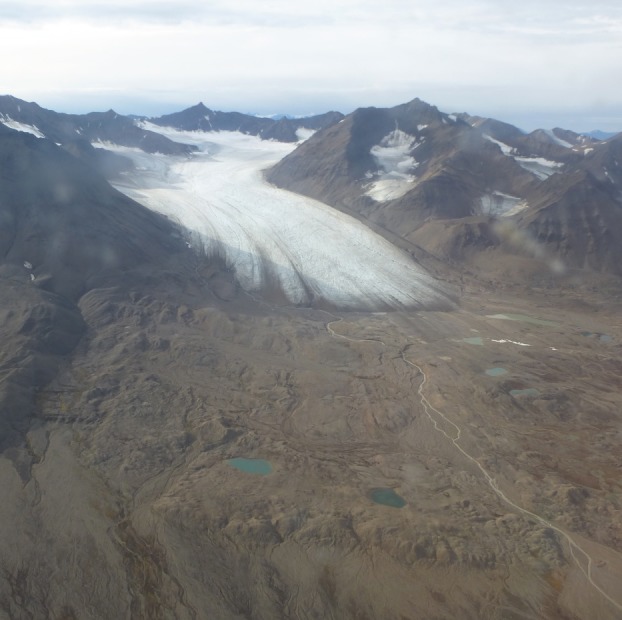
Aerial photograph of the forefield of Midtre Lovénbreen, a retreating valley glacier in Svalbard. For scaling purposes, the proglacial lakes vary between roughly 40–100 m in length. Photo credit: J. Bradley.

During recent decades, the cryosphere has received increasing recognition for harbouring diverse and active microbial communities [1]. Extremes in temperature, altitude, nutrient availability and seasonality create oligotrophic surroundings in which only highly specialized organisms can thrive. Glaciers and ice sheets at the poles and alpine regions have recently been subject to rapid changes in climate. The ‘Arctic amplification’ of near-surface air temperature has seen the Arctic warm at almost double the global average [2], along with earlier spring melting [3], milder winter days, and the retreat of snow and ice cover [4]. There has been a general volume decrease in Arctic glaciers and icecaps since about 1920 [4]. Retreating glaciers expose terrestrial ecosystems (figure 1) that have been previously locked under ice for thousands of years, providing unique environments to study primary colonization by simple cellular life. The fine glacial flour and highly reactive sediments found in recently deglaciated forefields may also have a consequence on global biogeochemical cycles and atmospheric CO_2_ concentrations, owing to the carbon sink associated with rock weathering [5]. Studies of plant colonization are fairly well established in glacial forefields [6–11]. However, studies based around microbes, the initial colonizers of glacial forefields, remain in comparatively early stages.

Soils at high latitudes and elevation develop over relatively long timescales, owing to low mean annual temperatures and slow weathering rates [12]. A chronosequence is a useful approach to gauge the development of forefield soils and the microbial communities associated with them over decadal timescales. By this method, taking a transect perpendicular to the snout of a receding glacier and using a space-for-time substitution, the development of recently exposed to established soils further from the ice-front can be characterized. This review covers the current body of work in deglaciated forefields in polar and alpine regions, and outlines suggestions for future research. Section 2 describes the major trends in the existing literature on forefield development, nutrient cycling and microbial communities. Section 3 considers the techniques employed in field studies to characterize soil microbial communities. Section 4 introduces the importance of seasonality of polar soils. Section 5 draws attention to model development and a greater understanding of the processes that dominate these ecosystems. Newly exposed glacier forefield ecosystems will become much more expansive with continued ice retreat in a warming climate. Hence it is imperative to understand and predict how these ecosystems will develop in the future.

## 2. Nutrient cycling in glacier forefields

**Figure 2.**
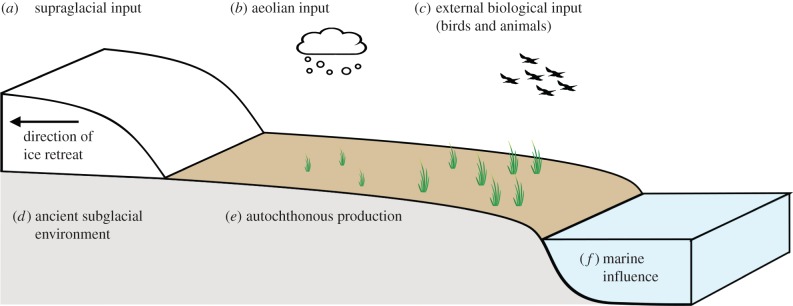
Pathways of nutrient cycling in a typical deglaciated forefield system.

It is widely regarded that microorganisms are the initial colonizers of recently exposed soils in deglaciated environments, such as in the Arctic [13]. Microbial life is considered fundamental in stabilizing soils and shaping the physical and biological development of these ecosystems [14]. Field studies have been conducted over a wide range of forefields, the majority of which are in alpine regions, but there are also examples from sub-polar and polar regions. Soil nutrient contents, rates of nitrogen fixation, enzymatic activity and respiration vary with stage of development, in turn altering the microbial community composition [13]. The major pathways of nutrients in a typical deglaciated forefield are outlined in figure 2. Allochthonous material is derived from (*a*) the glacier surface [15–17], (*b*) precipitation and aerial deposition [18,19] and (*c*) biological sources such as mammal and bird droppings [16]. Additionally, adjacent ecosystems such as (*f*) marine and (*d*) subglacial environments are likely to contribute to the nutrient dynamics [16,20–22]. Finally, (*e*) microbial activity within the forefield is considered a major contributor to nutrient cycling [13,23].

### (a) Carbon

**Figure 3.**
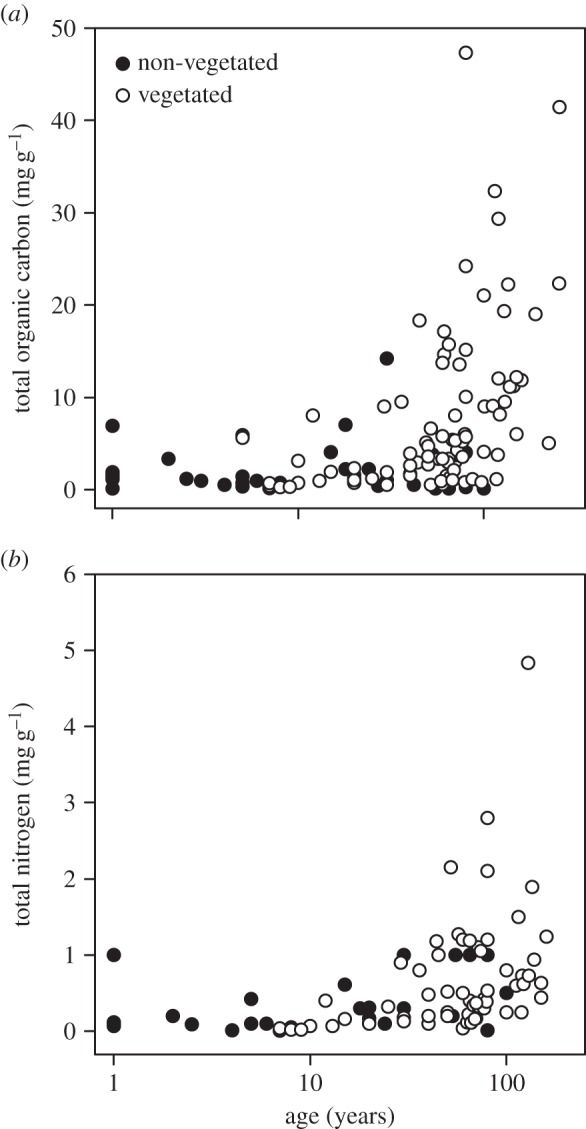
Accumulation of (*a*) total organic carbon and (*b*) total nitrogen in deglaciated forefield soils (see table 1 for source data).

The organic carbon content of glacial forefield soils is typically low, in the range of 0.1–40 mg g^−1^. This is thought to be an important control on the growth of biomass in these nutrient-poor ecosystems. Carbon content generally increases with age of soil, as biomass is established and biological activity increases [24]. This is illustrated in figure 3*a*, and comprises data from surface soils along 20 independent forefield studies (table 1) with comparable methodologies and units (discounting soils of 1000+ years in order to focus on the initial stages of succession). Carbon content positively correlates with soil age in all sites except two: the Larseman Hills, Antarctica [35], where no clear trend was observed along the transect and the Mendenhall Glacier (USA) [20] where there is a strong initial ancient and subglacial allochthonous carbon input which declines over the initial stages of succession.

**Table 1.** Source data for carbon, nitrogen* and phosphorus^*ɛ*^ content in deglaciated forefield chronosequences (figure 3).

field site	references
Athabasca Glacier, Canada	[6]
Rotmoosferner, Austria	[6]
Lyman Glacier, USA	[25]
Rotmoosferner, Austria*^,*ɛ*^	[26]
Ödenwinkelkees, Austria*^,*ɛ*^	[26]
East Brøgger Glacier, Svalbard*	[27]
Rotmoosferner, Austria*	[28]
Ödenwinkelkees, Austria	[29]
Puca Glacier, Peru*^,*ɛ*^	[30]
Damma Glacier, Switzerland*	[31]
Mendenhall Glacier, USA*^,*ɛ*^	[20]
Damma Glacier, Switzerland*	[32]
Damma Glacier, Switzerland*	[33]
Dongkemadi Glacier, China*	[34]
Larseman Hills, Antarctica*	[35]
Damma Glacier, Switzerland*	[36]
Damma Glacier, Switzerland	[24]
Robson Glacier, Canada*	[37]
Ecology Glacier, Antarctica*	[38]
Lys Glacier, Italy	[39]

The origin of carbon and other nutrients is often apparent in the chemical signature of the biological community and organic material. Studies on the Damma Glacier (Switzerland) indicate three distinct sources of carbon to initial soils: autochthonous primary production by autotrophic microorganisms, the deposition of allochthonous material (such as insects and soot particles) and ancient organic pools derived from under the glacier [13]. The balance between the autotrophic communities fixing their own carbon and dependence on external carbon sources to sustain microbial activity is crucial in shaping the overall forefield development and the associated biogeochemical cycles. Currently, from the existing body of research that encompasses multiple datasets and techniques, there is general disagreement in the dominant sources and fluxes of carbon in initial and developed forefield soils in different geographical regions.

Nutrient concentrations in initial soils are typically lower than developed soils (figure 3). Carbon producers such as cyanobacteria and eukaryotic microalgae form a rich source of organic matter, which in turn contributes carbon in nutrient-deficient soils. There is evidence for substantial autotrophic activity in initial soils at the Puca Glacier, Peru [23] based on a series of soil activity experiments. However, this autochthonous dominance is not reflected on other glaciers in polar and alpine regions. The initial soils of the Mendenhall Glacier (USA) [20] and Damma Glacier (Switzerland) [33] are subject to high allochthonous inputs, which are thought to be vital in sustaining microbial productivity. Glacier surfaces are also believed to be important in sustaining the productivity of downstream ecosystems by exporting labile organic matter, nutrients and inocula species via hydrological pathways [15,16]. For example, cryoconite melt is estimated to make up 13–15% of all meltwater runoff from Canada Glacier (McMurdo Dry Valleys, Antarctica) from which organic content is exported to downstream ecosystems and contributes to their productivity [17,40]. Similarly, outwash from subglacial environments is also likely to contribute to newly exposed soils in Svalbard [21,22].

Developed soils are typically richer in macronutrients such as carbon and nitrogen (figure 3). At the Damma Glacier (Switzerland), radio-isotope labelled carbon and *in situ* incubations have shown that carbon fluxes and microbial activity in developed soils are at least one order of magnitude greater than initial soils [24]. Microbial activity in developed soils in the Ödenwinkelkees Glacier forefield (Austrian Alps) [29] is sustained mostly by recalcitrant and ancient allochthonous carbon. This is in agreement with a study on the Robson Glacier forefield (Canada), where increasing phenol oxidase and peroxidase activities with age suggest that microbes in later successional stages are also using recalcitrant carbon resources as a dominant energy source [37].

### (b) Nitrogen

Nitrogen is commonly used in cellular synthesis of proteins and nucleic acid. The major sources of bioavailable nitrogen (nitrate, nitrite, ammonia and organic nitrogen) in forefield soils are microbially mediated fixation of atmospheric nitrogen gas (by cyanobacteria or some microbial groups associated with plant roots), internal remineralization and external sources, including snowmelt, aerial deposition and the breakdown of complex organic material [33]. Additionally, certain types of sedimentary and metasedimentary bedrocks may contain ecologically significant concentrations of nitrogen, which if liberated could impact biological nitrogen cycling in soils [41]. Typical nitrogen concentrations (total *N*) in deglaciated soils vary between 0.1 and 2 mg g^−1^ across studies listed in table 1, increasing with soil age (figure 3*b*). Unusually, Mendenhall Glacier (USA) shows the reverse trend owing to very high initial allochthonous substrate inputs [20]. Nitrogen contents of vegetated soils are typically higher owing to the contribution of plant litter and nitrogen-fixing microorganisms living symbiotically with plant roots [28,31,33,37].

There is general agreement between studies that microbially mediated nitrogen fixation is important in the initial stages of soil development. Nitrogen-fixing colonizers have been found to increase the accumulation of bioavailable nitrogen in soil and facilitate the colonization of later successional species in the Damma Glacier forefield (Switzerland) [31], Puca Glacier (Peru) [23,30], Mendenhall Glacier (Alaska) [20,42] and Anvers Island (Antarctica) [43]. However, it is also suggested that young soils at the Damma Glacier have very little potential for nitrogen fixation as low numbers of nifH gene copies associated with diazotrophs were found in initial soils [33]. Instead, the research suggests that initial communities acquire nitrogen mostly through allochthonous sources and the remineralization of ancient organic matter. Estimates for the natural deposition of nitrogen on the Damma Glacier are several orders of magnitude higher than nitrogen-fixation activity [33], suggesting that this allochthonous delivery may sustain nitrogen demand. Aerial deposition of bioavailable nitrogen also occurs in Svalbard as a result of western European pollution [18]. Studies in Austria, Alaska and Svalbard show that recalcitrant and ancient organic matter provide nitrogen, in accordance with findings on the Damma Glacier [13,20,29,33]. Microbially mediated denitrification has also been shown to occur in forefields, encouraged by anoxic conditions from a build-up of vascular plants and high-moisture soils [13,33,44,45].

### (c) Phosphorus

Phosphorus is a critical macronutrient for the growth and survival of living organisms, used in nucleic acids, ATP and phospholipids. Bioavailable phosphorus is usually abundant in the topsoil or bedrock of glaciated regions from weathering of the mineral surface. Thus, overall mineralogy of the area is likely to exert a strong control on biological activity in deglaciated soils. The bioavailability of phosphorus changes considerably along two deglaciated transects, the Hailuogou Glacier (Gongga Shan, China) and Damma Glacier (Switzerland) [46]. Initial soils on both forefields are depleted of bedrock-derived apatite-phosphorus and Al-bound phosphorus. However, acidification of developed soils (due to exudates from plant roots and the decomposition of organic matter) increases mineral dissolution and topsoil phosphorus status, which further facilitates the growth of microbial and plant communities in phosphorus-limited systems. Soil stocks of bioavailable phosphorus in four forefield systems show a general increase with chronosequence age from around 2 µg g^−1^ in undeveloped soils to around 8 µg g^−1^ in developed and vegetated soils [20,26,30].

## 3. Characterizing microbial communities in heterogeneous glacier forefields

Within the last decade, the development and commercialization of genetic sequencing techniques has enabled researchers to carry out much more detailed analyses of microbial communities in the environment. In 2002, DNA extraction and amplification was used to indicate a difference in bacterial community composition in glacial forefields in Switzerland [47], showing for the first time that diverse microbial communities inhabit even the least developed soils. Since, increasing availability and decreased cost of molecular techniques has seen their wide use in characterizing microbial community development in glacial forefields [20,23,30,34–36,38,42,48–50]. Cyanobacteria, Proteobacteria, Actinobacteria and various species of fungi are commonly found in deglaciated soil ecosystems in the Arctic and Antarctic [9,20,21,35,43]. Although far less studied than the other microbial groups, Archaea have also been found in glacial forefields [50]. Numerous studies have shown the abundance of gene copies relating to nitrogen fixation and mineralization [31,33,43] and denitrifying bacteria [28,51]. Combinations of DNA barcoding, RNA amplification and extensive biogeochemical analysis of the soil environment have allowed scientists to robustly determine the functional traits of the microbial communities and their ability to metabolize a wide range of substrates as energy sources. Next-generation metagenomic technology has recently been used to characterize the biological components of glacial sediments [52] and Antarctic soils [53], revealing a much greater diversity of lineages and functions than previously thought. However, using metagenomic technology in polar environments is particularly challenging because of the relatively low concentrations of microbial biomass, resulting in low recoveries of quality genomic DNA, particularly in young soils.

Bacteria and fungi exhibit different successional patterns during primary colonization. For example, at the Lyman Glacier (USA), bacterial communities appeared to converge towards single community types, whereas fungi (which are more dependent on fixed carbon and nitrogen and typically colonize at a later stage) did not show evidence of convergence [54,55]. Increasing microbial diversity in developed soils broadens the pathways of litter decomposition, owing to enhanced enzymatic capabilities for degrading complex substrates, and higher functional niche complementarity [56]. Nearly, one-third of carbon stocks from the Damma Glacier forefield (Switzerland) was lost to microbial respiration in developed soils, indicating a highly active community of decomposers [56]. Microbial productivity is also determined by the quality of organic substrate, indicated by a build-up of poor quality recalcitrant carbon in older soils of the Damma Glacier resulting in decreased availability of soil organic matter with age [32]. Differences in Archaeal community composition have been observed in the Damma Glacier, where there is a shift from Euryarchaeota in young soils to Crenarchaeota in old soils [50]. The presence of Euryarchaeota in young soils, which have a number of known methanogenic representatives, could indicate a strong influence of subglacial microbial communities and biogeochemical functions in the initial stages of soil succession.

The forefield of a receding glacier is extremely heterogeneous in terms of physical landforms, soil structure and environmental conditions, each of which directly impact the composition, activity and function of the microbial community. A key assumption of the chronosequence approach is that each site along the chronosequence was subject to the same initial conditions and followed the same sequence of change. The extent to which this is true for many field-sites is questionable, as glacier forefields are subject to large fluctuations in climate and hydrology over decadal timescales. Heterogeneity exists across multiple spatial scales. For example, soil rizospheres at the Damma Glacier (Switzerland) were found to be two to six times richer in macronutrients than bulk soils [31]. The biogeochemical signature of the soil is not just the result of a single microbe but the whole microbial community. Temporal heterogeneity also affects chronosequence studies. Single evaluations of a soil chronosequences are snap-shots that are likely to change in the following days to weeks depending on hydrology and local environmental factors. Landscape mineralogy also exerts a significant control on the microbial community structure indicated by the clear difference between calcareous soils and siliceous soils in two alpine forefields, despite negligible differences in macronutrient concentrations between sites [57]. Consequently, comparing and contextualizing different chronosequence studies remains challenging, with multiple factors playing a role in the stages of soil development.

## 4. Seasonality of a glacier forefield ecosystem

Although the year-on-year development of forefield ecosystems is increasingly well studied, very few investigations consider the winter dynamics. Polar winters are characterized by sub-zero temperatures, periods of 24 h darkness (in high latitudes), and snow cover. Microbial activity during the winter has long been assumed to be insignificant to forefield ecosystem dynamics as microbial populations lie dormant under adverse environmental conditions. However, during winter, overlying snowpacks may insulate the soils and protect soil organisms from frost damage [58]. As little as 30 cm of snow is sufficient to decouple soil and air temperatures thus promoting survival of microbial communities [59]. In the Arctic, earlier snow cover results in higher minimum soil temperatures, keeping soils unfrozen for much of the winter [60–63]. Active microbial nitrogen cycling occurs in winter snowpacks in Svalbard [19]. Biologically available nitrogen in the spring melt is then assimilated into the underlying soils and incorporated as organic nitrogen [64–66]. However, gas exchanges between the soil and atmosphere may be limited by thick snow cover and periodic melting causing ice-encasement, leading to anoxia and an accumulation of CO_2_ [67], resulting in microbially induced denitrification and N_2_O emissions [44,68].

Winter soils in various alpine environments harbour an active microbial community of decomposers that continue to respire CO_2_ [63,69–71]. Ongoing activity is fuelled by fungal and microbial decomposition of organic polymers and phenolic compounds [72,73]. Temperature is a probable driver of microbial processes and community development. Over winter, there are distinct community shifts towards cold-adapted fungi and decomposers such as Actinobacteria in alpine and Antarctic tundra soils [72–74]. As such, the microbial community typically sampled during summer may not be representative of the year-round variability that the natural system experiences.

The onset of spring melt causes changes to the hydrological and biological regime of the forefield system. Rapid solute efflux due to preferential elution has the potential to export significant quantities of solute labile carbon to unfrozen soils via infiltration [62]. However, if soils remain frozen at the time of snowmelt, infiltration is prevented and a significant proportion of nutrients may be lost owing to wash-out [62,75]. A continuous snowpack promotes the accumulation of unfrozen soil water, solutes and microbial transformations of carbon, nitrogen and phosphorus, whereas an intermittent snowpack and pulses of water encourages leaching of soluble nutrients, and redox reactions [62]. This is likely to have a significant effect on the annual delivery of nutrients to forefield soils as a result of spring melt.

Seasonal climate variations as a result of anthropogenic warming [4] will undoubtedly affect the development of microbial communities in forefield soils. Bacterial activity is likely to increase with longer growing seasons [76]. However, carbon loss from the soil may be accelerated by warming temperatures [77]. Changes to the hydrological regime may cause extra disturbance to soil communities [3], while a reduction in snow cover may also hinder biological development owing to the loss of a protective insulating layer and exposure to frost damage [58]. It is imperative that the distinct seasonal changes which polar regions experience are captured in studies on forefield studies, since it is likely to have a direct impact on the microbial community structure, nutrient cycling and long-term development of the soil.

## 5. Numerical modelling of the forefield ecosystem

Typical field and laboratory methodologies have yielded volumes of data related to geochemical and molecular information. When combined with numerical modelling tools, the underlying processes controlling the system dynamics can be quantitatively evaluated to provide indication of the potential sensitivity of the system to environmental changes.

Incorporating models into studies of microbial succession is becoming more feasible as our understanding deepens alongside increased computational power and model development [78]. Modelling requires an understanding of the fundamental processes and is driven by data. Process-based modelling of microbial ecology, whereby the most important biogeochemical and physical processes are modelled explicitly, has gained popularity in a range of soil and sediment ecosystems [79–83]. Process-based models have successfully described nitrogen turnover in soils [84], nutrient fluxes in Arctic soils [81,82] and litter degradation in a temperate environment [83].

As increasing data accumulates from fieldwork, it will become more apparent which level of model complexity is required to adequately represent microbial succession in forefield soils. Ultimately, models should be designed to answer the most pressing questions as accurately and with as much confidence as possible. In forefield ecosystem dynamics, models could be used to explore such unknowns as: (i) the relative importance of allochthonous and autochthonous nutrient sources (such as nitrogen input with snowmelt) in determining the microbial community, (ii) quantifying the effect of disturbances, (iii) assessing how microbial diversity influences soil development, (iv) the importance of seasonality, (v) the sensitivity of chronosequence development to future climate change, and (vi) identifying gaps in our understanding to inform future fieldwork and research questions. To fit these purposes, models must have an explicit representation of microbial community dynamics and their interactions with major nutrient pathways and changing environmental conditions.

There are unique problems associated with applying existing modelling principles to forefield soil development. For example, growing seasons are punctuated by harsh winter conditions. Therefore, seasonality must be resolved to accurately portray the progression of one growing season to the next. Decomposer activity would be seasonally variable [24] but is often assumed to be constant in shorter time-frame models [85]. The majority of datasets do not account for seasonality, representing only summer. In a carbon enrichment experiment at the Damma Glacier (Switzerland) tracking respiratory losses from soils, it was estimated that between 62 and 72% of annual CO_2_ effluxes were the result of respiration during a four-month long summer period [24,56], suggesting that respiration continued over winter. Additionally, there is potential for significant errors in the discrepancies between the scale at which microbial ecosystems are present in the soils (less than 10^−3^ cm), the scale at which they can be sampled (1–10 cm), and scales at which they are modelled. Processes that dominate at the microscopic scale must be re-parametrized so that they are applicable on a coarser spatial scale. This upscaling is unlikely to respond in a linear fashion [86] and may lead to uncertainties.

In biogeochemical soil models, parameters are usually calibrated using empirical field data [81,83,84,87,88]. However, it is often difficult to isolate the effects of specific variables to determine realistic parameter values, since microbial activity in a glacier forefield is simultaneously affected by temperature, nutrient availability, light and moisture availability [16]. Environmental factors can be controlled to a large extent in laboratory incubations, thereby isolating single variables and thus quantifying the sensitivity of the system to specific manipulations. Thus, model parameters can be informed by laboratory studies, such as nitrogen turnover dynamics in soils [89] and temperature sensitivity of microbial growth rates [90]. Representing unknown and unquantifiable components in a model description often introduces errors, uncertainties and unrealistic parameter values; however, these problems can also result from over-simplification. Models describing forefield development must ultimately be a simplified version of the system, well constrained by observational data, without sacrificing the components that are essential for its understanding.

## 6. Conclusion and future outlook

The Arctic and Antarctic regions are warming at double to triple the global average rate [2,91]. Thus, it is likely that deglaciated forefields will become much more expansive in the future as a result of continued ice retreat. It is important to understand the dominant controls on ecosystem development to determine long-term productivity and understand how landscapes become colonized and productive. Simple descriptions of species distribution and environmental biogeochemistry are progressing onto a deeper understanding of the processes which drive the spatial and temporal patterning of microbial communities, and establishing the dominant controls on their growth, activity and succession.

Over the last decade, there has been increasing interest in attempting to characterize forefield development in relation to microbial community establishment and nutrient cycling. Autotrophic microorganisms are responsible to some extent for the build-up of initial pools of carbon in the soils [23]. Similarly, nitrogen-fixing species may facilitate later colonization of the soil by increasing the overall nitrogen bioavailability [20,23,30,31,42,43]. However, it still remains unclear to what extent microbial life is responsible for the initial build-up of nutrients, compared to external sources. To further appreciate how forefield ecosystems are connected in the cryosphere and biosphere, detailed understanding of the delivery mechanisms, pathways and export of allochthonously derived and autochthonously produced nutrients is needed. The seasonal dynamics of these ecosystems are also largely unexplored. Projected warming of polar regions is most prominent during the winter months in the Arctic; therefore, it is increasingly important to study winter dynamics of soils. Yet, few studies currently incorporate seasonality into their sampling strategy or analysis. Finally, the development of numerical models which test the importance of external nutrient loading and seasonal variation may be able to provide answers to the most pressing questions. Model building helps us learn more about the general functioning of these systems, and may be able to guide future research and the design of field experiments. Global climate change not only results in a transformation of the physical landscape due to melting and retreating ice masses, but also rapid changes in biogeochemical cycles. Deglaciated forefields are ideal locations to study such changes.

Future progress will largely be dependent on the increased availability of year-round observational data from a range of forefields, as well as efforts to quantitatively evaluate the importance of various processes and external forcings. This will enable some predictive capability, and a better mechanistic understanding of the underlying processes which drive microbial community development in forefield soils, for both small-scale glacier systems and large-scale ice sheet retreat.
